# Self-Reported Caring Behaviors, Structural Empowerment, and Psychological Empowerment Among Nurses: A Cross-Sectional Study

**DOI:** 10.3390/healthcare14213634

**Published:** 2026-10-27

**Authors:** Samirh Said Alqhtani

**Affiliations:** 1 College of Nursing, King Saud bin Abdulaziz University for Health Sciences, Jeddah P.O. Box 3660, Saudi Arabia; alqahtanis@ksau-hs.edu.sa; 2 King Abdullah International Medical Research Center, Jeddah P.O. Box 9515, Saudi Arabia; 3 Ministry of the National Guard—Health Affairs, Jeddah 22384, Saudi Arabia

**Keywords:** caring behaviors, structural empowerment, psychological empowerment, nurses

## Abstract

**Background/Objectives**: Caring behaviors are central to professional nursing practice and the delivery of high-quality, patient-centered care. Structural empowerment concerns nurses’ access to information, resources, support, and opportunities, whereas psychological empowerment reflects meaning, competence, self-determination, and impact. Although structural and psychological empowerment have been associated with favorable nursing and organizational outcomes, the moderating role of psychological empowerment in the relationship between structural empowerment and self-reported caring behaviors remains underexplored. This study aimed to determine whether psychological empowerment moderates the relationship between structural empowerment and self-reported caring behaviors among nurses in Saudi Arabia. **Methods**: A cross-sectional study was conducted among 206 nurses. Data were collected using validated self-reported measures. Analyses included descriptive, correlation, covariate-adjusted hierarchical regression, sensitivity analyses, and secondary nonlinear and exploratory mediation analyses. **Results**: Structural empowerment was positively associated with self-reported caring behaviors (r = 0.38, *p* < 0.001) and psychological empowerment (*r* = 0.52, *p* < 0.001). Psychological empowerment was strongly correlated with self-reported caring behaviors (*r* = 0.69, 95% CI: 0.61–0.76, *p* < 0.001) and remained positively associated after adjustment (B = 0.49, 95% CI: 0.38–0.60, *p* < 0.001). The adjusted interaction provided insufficient evidence for hypothesized moderation (B = −0.022, 95% CI: − 0.046 to 0.001, *p* = 0.066), consistent with the sensitivity analyses. Secondary analyses identified a nonlinear psychological empowerment- self reported caring behaviors association and an exploratory indirect association through psychological empowerment (indirect estimate = 0.066; bootstrap 95% CI, 0.045–0.091). **Conclusions**: Structural empowerment and psychological empowerment were positively correlated with self-reported caring behaviors among nurses. Evaluating the effectiveness of interventions aimed at psychological empowerment requires longitudinal or experimental research.

## 1. Introduction

Caring is a central component of professional nursing practice and an essential element of patient-centered care. It is conceptualized as a relational, ethical, and professional commitment that extends beyond technical competence to include emotional presence, empathy, and advocacy [1]. Watson’s Theory of Human Caring views caring as a relational process through which nurses establish meaningful therapeutic relationships while attending to patients’ physical, emotional, and psychosocial needs [1]. Previous studies suggest that caring behaviors are associated with greater patient satisfaction and higher perceived quality of care [2,3]. However, nurses’ ability to consistently demonstrate caring behaviors may be influenced by the organizational environment in which care is delivered [4].

Empowerment has been widely examined in nursing practice. Empowerment has two perspectives: structural empowerment (SE) and psychological empowerment (PE). Kanter’s Theory of Structural Empowerment (1977) provides a useful framework for understanding how the work environment can support nurses’ professional practice [5]. According to Kanter, employees are more likely to function effectively when they have access to information, resources, support, and opportunities for growth and development [5]. In nursing, SE reflects nurses’ access to organizational structures that enable them to perform their roles effectively. In Saudi Arabia, nurses have reported a moderate level of empowerment and a significant positive relationship between empowerment and organizational commitment [6]. Structurally empowered work environments have been associated with lower burnout and turnover intentions, stronger professional practice, and better patient and organizational outcomes [7,8,9]. Such environments may provide nurses with the support, resources, and professional autonomy needed to engage meaningfully with patients and demonstrate caring behaviors. However, structural conditions may not fully explain how nurses behave in clinical practice. Therefore, PE extends the organizational perspective by focusing on how individuals perceive and experience their work.

PE refers to an individual’s psychological experience of empowerment at work, including perceptions of control, competence, and capability in the workplace [10]. Spreitzer conceptualizes PE as an intrinsic motivational state reflecting how individuals perceive their roles within the workplace [11]. It comprises four dimensions: meaning, competence, self-determination, and impact [11]. Meaning refers to the value individuals attach to their work; competence reflects confidence in their ability to perform effectively; self-determination represents autonomy and choice in carrying out work activities; and impact represents the perceived ability to influence work and organizational outcomes [11]. PE has been associated with work engagement, resilience, job satisfaction, and professional commitment among nurses [12,13,14]. Higher workplace empowerment has also been associated with increased work motivation and reduced occupational stress among nurses [15].

SE and PE have been positively associated with organizational commitment and safety performance [16]. SE focuses on the delegation of decision-making authority, the transfer of power to employees and increased employee involvement, whereas PE focuses on employees’ perceptions of empowerment and intrinsic motivation [17]. Building on this distinction, PE was proposed as a moderator to examine whether the association between SE and self-reported caring behaviors varies according to nurses’ level of PE. Nurses with similar access to organizational opportunities and resources may differ in how they use these conditions in caring practice, depending on their perceived meaning, competence, self-determination and impact. This possibility motivated H4, which proposed that PE conditions the strength of the SE-caring behaviors association. This represents a theory-informed conditional-association hypothesis rather than an interaction directly established by the cited studies. Previous research has also examined indirect relationships through PE [16], making mediation a plausible alternative. Moderation remained the primary analysis, while an exploratory mediation analysis additionally examined whether PE statistically accounted for the association between SE and self-reported caring behaviors.

**Figure 1:**
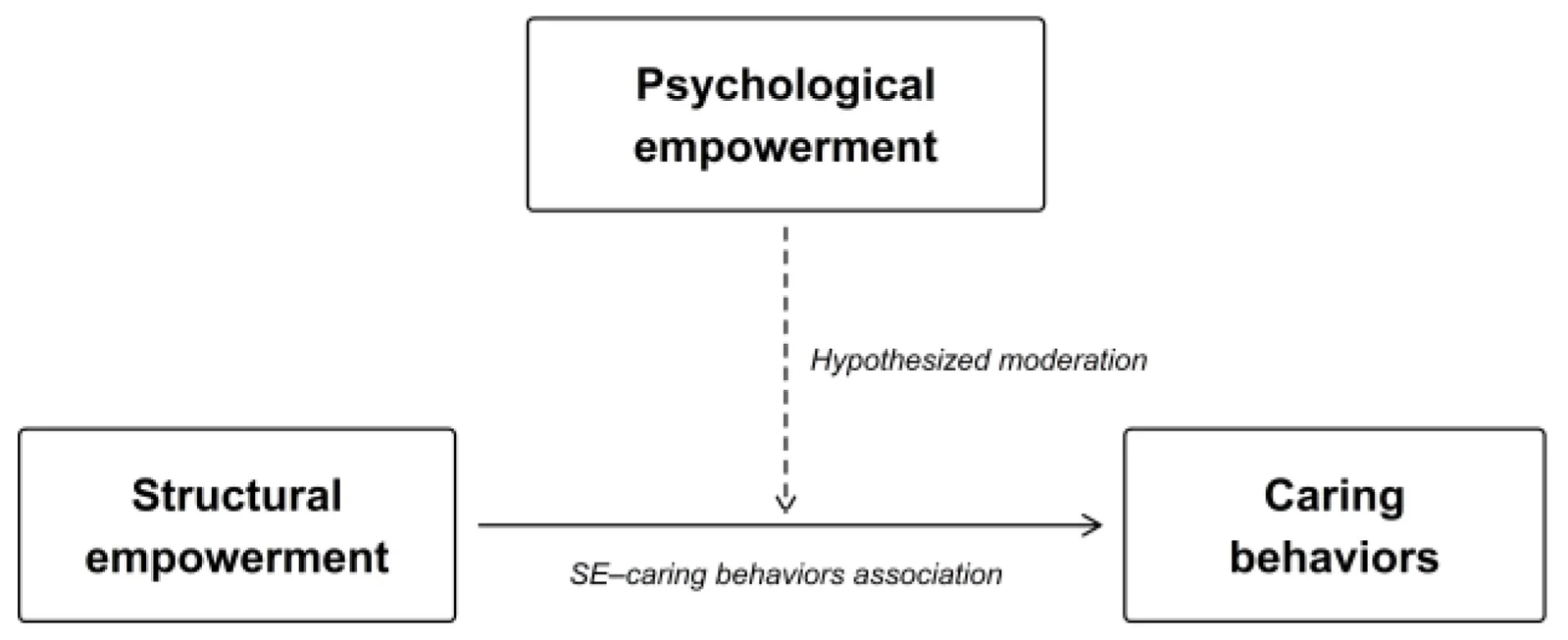
Conceptual Model of PE as a Moderator of the Association Between SE and Self-Reported Caring Behaviors Among Nurses. The solid arrow represents the hypothesized association between SE and self-reported caring behaviors. The dashed arrow indicates the proposed moderating role of PE. The diagram presents the study hypothesis rather than an established causal relationship or confirmed moderation effect.

Kanter’s Structural Empowerment Theory, Spreitzer’s Model of Psychological Empowerment, and Watson’s Human Caring Theory inform the conceptual framework of this study. Kanter’s theory describes access to organizational opportunities, information, support, and resources, whereas Spreitzer’s model explains nurses’ psychological responses to their work environment. Watson’s theory provides a relational perspective for understanding caring in nurse–patient interactions. Together, these frameworks inform the proposed relationship but do not directly establish PE as a moderator (Figure 1). The present study examined the proposed conditional association; however, its cross-sectional design cannot establish temporal ordering or determine which mechanism best explains the observed relationships.

Although structural and psychological empowerment have been associated with several nursing outcomes, whether PE moderates the relationship between SE and self-reported caring behaviors warrants further investigation. Therefore, this study aimed to examine the relationships among SE, PE, and self-reported caring behaviors and to test whether PE moderates the association between SE and self-reported caring behaviors among nurses in Saudi Arabia.

The following hypotheses were proposed based on Kanter’s Theory of Structural Empowerment, Spreitzer’s Model of Psychological Empowerment, and Watson’s Theory of Human Caring.

**Hypothesis** **H1.**  *SE is positively associated with self-reported caring behaviors among nurses.*

**Hypothesis** **H2.**  *PE is positively associated with self-reported caring behaviors among nurses.*

**Hypothesis** **H3.**  *SE is positively associated with PE among nurses.*

**Hypothesis** **H4.**  *PE (moderating variable) moderates the relationship between SE (independent variable) and self-reported caring behaviors (dependent variable) among nurses.*

## 2. Materials and Methods

### 2.1. Study Design and Participants

A descriptive cross-sectional correlational design was used to examine the relationships among the study variables at a single point in time, providing an efficient and cost-effective approach to data collection [18]. Participants were recruited using a convenience sampling technique based on their eligibility, availability, and willingness to participate in the study [19]. The study was conducted in a single tertiary hospital in Riyadh, Saudi Arabia, within a larger healthcare system. At the time of the study, the hospital had 1973 operational beds and provided care across medical, surgical, critical care, emergency, obstetric/gynecological, pediatric, and outpatient services. Nursing services operated through a formal governance structure, with executive nursing leadership and unit-level management overseeing clinical standards, nursing quality indicators, and professional development. Participants were selected according to predefined inclusion and exclusion criteria. Registered nurses who provided direct patient care in clinical settings and agreed to participate were eligible for inclusion. Nurses working exclusively in administrative roles without a direct patient care role, as well as nursing interns and students, were excluded.

### 2.2. Sample Size

Sample-size planning considered precision in estimating the mean scores of SE, PE, and self-reported caring behaviors, together with the planned correlation and hierarchical regression analyses. For mean estimation, the formula n = Z^2^σ^2^/d^2^ was applied. An assumed standard deviation of 1.0 was obtained using the range approximation (7 − 1)/6 [20]. With a 95% confidence level and a margin of error of 0.15, the required sample was 171 participants. For the three planned Pearson correlations, G*Power version 3.1.9.7 was used with a two-sided test, an expected correlation of r = 0.30, a null correlation of zero, 80% power, and a Bonferroni-adjusted α of 0.0167. This calculation required 112 participants.

Separate calculations were conducted for the three stages of the hierarchical regression analysis using the F-tests family in G*Power, α = 0.05, and 80% power. For the covariate-only model (Model 1), the fixed-model R^2^-deviation-from-zero test was specified. A moderate overall effect of f^2^ = 0.15 and a conservative allowance of up to 23 covariate coefficients yielded a required sample of 166 participants. For Model 2, the fixed-model R^2^-increase test assessed the additional variance explained by adding structural and psychological empowerment. With two tested predictors, 25 total predictors, and a small-to-moderate incremental effect of f^2^ = 0.05, the required sample was 197 participants.

For Model 3, the fixed-model R^2^-increase test assessed the additional variance explained specifically by the SE × PE interaction. The calculation included one tested predictor, 26 total predictors, f^2^ = 0.05, α = 0.05, and 80% power, yielding a required sample of 160 participants. For both incremental tests, the effect size was defined as f^2^ = (R^2^full − R^2^reduced)/(1 − R^2^full). The largest requirement across the planned analyses was 197 participants, which was exceeded by the recruited sample of 206 nurses.

### 2.3. Data Collection

After obtaining IRB approval, the Microsoft Forms link was sent via email to the head of the nursing department. The head of the nursing department subsequently distributed the survey link by email to eligible nursing groups. The number of nurses who received the invitation and the number of questionnaires initiated but not submitted were not recorded; therefore, a response rate could not be calculated. Two questionnaires were excluded because participants did not consent to participate, leaving 206 participants for analysis. Only the researcher had access to the responses. To minimize perceived pressure to participate, the invitation emphasized that participation was voluntary, and that declining to participate would have no consequences. Nursing management had no access to individuals’ responses or information identifying participants, and participants completed the survey independently. The survey began with a cover page providing information about the study purpose and informed consent, followed by demographic data and the validated study instruments. Data were collected from May 2026 to July 2026. The survey required approximately 8 to 10 min to complete, and no personally identifiable information was collected. The survey settings were configured to allow only one response per participant to minimize duplicate submissions.

#### Measurements

A. The demographic data included gender, age, marital status, educational level, years of experience, work position, and clinical unit. The original English-language instruments were used; no translation or cultural adaptation was undertaken for this study, because the participating nurses used English in professional communications.

B. The Caring Behaviors Inventory-16 (CBI-16) is a 16-item instrument used to assess self-reported caring behaviors and is grounded in Watson’s Theory of Human Caring [21]. Items are rated on a 6-point Likert scale ranging from 1 (Never) to 6 (Always). Item scores can be summed to produce a total score ranging from 16 to 96. In this study, self-reported caring behaviors were analyzed using the overall mean of the 16 items, yielding a score ranging from 1 to 6, with higher scores indicating higher levels of perceived caring behaviors [21]. The CBI-16 demonstrated excellent internal consistency, with a Cronbach’s alpha of 0.95 [22]. The CBI-16 has also demonstrated satisfactory construct validity through exploratory and confirmatory factor analyses [21].

C. The Conditions for Work Effectiveness Questionnaire-II (CWEQ-II) is a 19-item instrument used to assess SE [23]. It consists of six subscales reflecting access to opportunity, information, support, resources, formal power, and informal power. Each subscale consists of three items, except informal power, which contains four items. Responses are on 5-point Likert scales. A mean score ranging from 1 to 5 is calculated for each subscale, and the six-subscale means are summed to obtain a total SE score ranging from 6 to 30, with higher scores indicating greater perceived SE [23]. Scores ranging from 6 to 13 indicate low empowerment, 14 to 22 indicate moderate levels, and 23 to 30 indicate high empowerment [23]. Two additional global empowerment items were administered as a separate validity check measure and scored by averaging their responses. These items were scored separately and excluded from the six-subscale SE total. The CWEQ-II has demonstrated good internal consistency with Cronbach’s alpha of 0.89. Its construct validity has been reported in nursing research [24].

D. The Spreitzer Psychological Empowerment Scale comprises 12 items across four dimensions: meaning, competence, self-determination, and impact [11]. Each dimension consists of three items rated on a 7-point Likert scale ranging from 1 (very strongly disagree) to 7 (very strongly agree) [11]. Item scores can be summed to produce a total score ranging from 12 to 84. The scale demonstrated strong internal consistency reliability, with Cronbach’s alpha coefficients typically ranging from 0.72 to 0.94 across dimensions. The scale has been used across diverse occupational settings, including nursing populations, and has consistently demonstrated acceptable reliability and validity [11,25].

### 2.4. Ethical Consideration

The study was conducted in accordance with the Declaration of Helsinki and was approved by the Ethics Committee of King Abdullah International Medical Research Center (KAIMRC) (Institutional Review Board [IRB] approval number: 00000336626; study reference number: NRR26/051/4). Electronic informed consent was obtained from all participants before participation. The consent form provided information about the purpose of the study, emphasized the voluntary nature of participation, and informed participants of their right to withdraw at any time. Participants’ privacy and the confidentiality of their information were maintained throughout the study.

### 2.5. Data Management and Analysis Plan

Categorical variables were summarized using frequencies and percentages, and continuous variables using means, standard deviations, medians, and interquartile ranges. Internal consistency was evaluated using Cronbach’s alpha with bootstrap 95% confidence intervals; the Spearman–Brown coefficient was used for the two-item global empowerment score. Pearson correlations examined the three planned associations among SE, PE, and self-reported caring behaviors, with Bonferroni-adjusted *p*-values. Spearman correlations were conducted as sensitivity analyses.

The hypothesized moderation model is presented in Figure 1. Hierarchical ordinary least-squares regression was conducted with self-reported caring behaviors as the outcome. Model 1 included the prespecified covariates of gender, nursing experience, marital status, nursing education, and clinical unit. These covariates were selected based on theoretical and contextual relevance rather than univariable statistical significance. Model 2 added mean-centered SE and PE, and Model 3 added the SE × PE interaction. Unstandardized coefficients, 95% confidence intervals, *p*-values, R^2^, adjusted R^2^, and changes in R^2^ were reported. HC3 robust Wald tests evaluated the overall models and the predictors added at each hierarchical step.

Regression diagnostics included residual-versus-fitted, normal Q–Q, scale-location, leverage, and Cook’s distance plots, supplemented by formal assessments of heteroskedasticity, functional form, residual normality, and multicollinearity (Supplementary Table S1 and Supplementary Figure S1). Because the residual variance was nonconstant, HC3 heteroskedasticity-robust standard errors were used for the primary regression inference. Potential nonlinearity was examined in a secondary adjusted model that added squared centered SE and PE terms while retaining the main effects, interaction, and covariates. The quadratic terms were evaluated jointly using an HC3 robust Wald test. The adjusted PE–caring behaviors relationship was visualized using average model predictions across participants’ observed SE values and covariate profiles, restricted to the observed PE range. Additional sensitivity analyses included fractional-logit regression for the bounded self-reported caring behaviors outcome and exclusion of observations with Cook’s distance greater than 4/N (Supplementary Table S2).

An exploratory mediation analysis examined whether PE statistically accounted for the association between SE and self-reported caring behaviors. The analysis estimated the SE–PE association (path a), the PE–caring behaviors association controlling for SE (path b), the total SE–caring behaviors association (path c), and the direct association after accounting for PE (path c′). All models included the same covariates as the hierarchical regression. The indirect effect was calculated as a × b, with a percentile-bootstrap 95% confidence interval based on 5000 participant resamples. Because all variables were measured concurrently, the mediation analysis was considered exploratory and was not interpreted as establishing temporal or causal effects.

No item responses were missing in the final analytic dataset; therefore, no imputation or exclusion due to incomplete item responses was required. Analyses used complete cases and two-sided tests, with *p* < 0.05 considered statistically significant. Except for the three planned correlations, *p*-values were not adjusted for multiple testing. Analyses were conducted using R version 4.2.2, primarily using dplyr for data preparation, psych for reliability analysis, sandwich for HC3 covariance estimation, boot for bootstrap analysis, and ggplot2 for figures and diagnostic plots.

## 3. Results

### 3.1. Participant Demographic and Professional Characteristics

The sample comprised 206 nurses, of whom 174 (84.5%) were female. The mean age was 32.43 years (SD, 9.23), with a range of 17–60 years. More than half were single (53.9%), and 78.6% held a bachelor’s degree in nursing. Mean nursing experience was 8.25 years (SD, 7.81), with a range between 0.67–41 years. Almost half worked in medical units (49.0%), followed by critical care and emergency (31.1%), outpatient/clinic settings (11.2%), and surgical wards (8.7%). No data were missing for the demographic and professional characteristics reported in Table 1.

#### 3.1.1. Distributions and Internal Consistency of the Study Measures

Participants’ scores were available for all 206 nurses (Table 2). The SE six-subscale total averaged 20.71 (SD, 4.24), with a median of 20.54 (IQR, 18.00–23.40). Opportunity had the highest subscale mean (3.81), and formal power had the lowest (3.05); global empowerment averaged 3.34 (SD, 1.03). PE averaged 5.38 (SD, 1.03), with a median of 5.42 (IQR, 4.60–6.08). Meaning and competence had the highest dimension means (both 5.65), followed by self-determination (5.18) and impact (5.06). Self-reported caring behaviors averaged 5.26 (SD, 0.81), with a median of 5.56 (IQR, 4.94–5.88).

**Figure 2:**
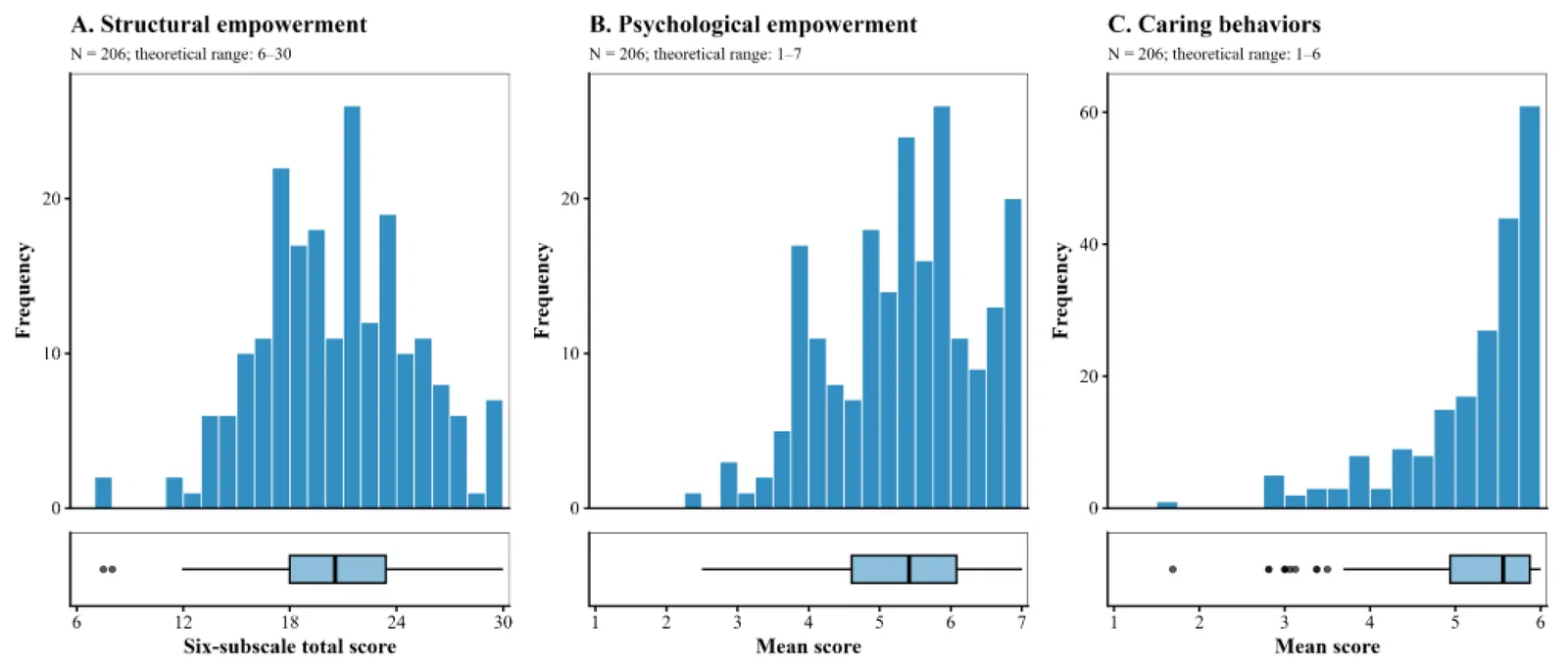
Distributions of SE, PE, and self-reported caring behaviors among 206 nurses. Histograms and corresponding boxplots show SE six-subscale total scores (**A**), PE mean scores (**B**), and caring behaviors mean scores (**C**). Horizontal axes display the full theoretical ranges of 6–30, 1–7, and 1–6, respectively. Boxes indicate the interquartile range, central lines represent medians, and whiskers extend to the most extreme observations within 1.5 interquartile ranges of the box limits. Points beyond the whiskers represent observations outside these limits.

Figure 2 shows upper-end clustering and a longer lower tail for self-reported caring behaviors, with less pronounced asymmetry in the empowerment measures. Cronbach’s α was 0.93 (95% CI, 0.91–0.94) for the 19 SE items, 0.93 (95% CI, 0.92–0.95) for PE, and 0.95 (95% CI, 0.93–0.96) for self-reported caring behaviors. Subscale α coefficients ranged from 0.77 to 0.89 for SE and from 0.82 to 0.85 for PE. The two-item global empowerment measure had a Spearman–Brown coefficient of 0.90 (95% CI, 0.84–0.94).

#### 3.1.2. Associations Among Study Measures and Participant Characteristics

Table 3 shows that all three planned associations were positive and statistically significant after Bonferroni adjustment, all adjusted (*p* < 0.001). SE correlated with self- reported caring behaviors (*r* = 0.38; 95% CI, 0.25–0.49) and PE (*r* = 0.52; 95% CI, 0.41–0.61). The largest correlation was between PE and self-reported caring behaviors (r = 0.69; 95% CI, 0.61–0.76). Spearman sensitivity analyses yielded similar results, with corresponding coefficients of 0.40, 0.56, and 0.65 (all Bonferroni-adjusted *p* < 0.001). In exploratory univariable analyses using conventional OLS inference, male nurses reported lower self- reported caring behaviors scores than female nurses (B = −0.46; 95% CI, −0.76 to −0.16; *p* = 0.003), whereas nursing experience was positively associated with self- reported caring behaviors (B = 0.016 per year; 95% CI, 0.002–0.030; *p* = 0.026). Associations with age, marital status, nursing education, and clinical unit were not statistically significant (Supplementary Table S3). Associations with age, marital status, nursing education, and clinical unit were not statistically significant (*p* > 0.05).

### 3.2. Hierarchical Regression Analysis of Caring Behaviors

The covariate-only model explained 10.2% of the variance in caring behaviors (adjusted R^2^ = 0.060), although the covariates were not jointly significant (HC3 robust Wald F [9, 196] = 1.40; *p* = 0.191). Within this model, male nurses reported lower caring behaviors scores than female nurses (B = −0.47; 95% CI, −0.86 to −0.07; *p* = 0.020), whereas nursing experience was positively associated with caring behaviors (B = 0.02 per year; 95% CI, 0.01–0.04; *p* = 0.007). Adding structural and PE in Model 2 increased the explained variance by 41.37 percentage points (ΔR^2^ = 0.4137), and the added predictors were jointly significant (HC3 Wald F [2, 194] = 65.40; *p* < 0.001). Model 2 explained 51.5% of the variance in caring behaviors (adjusted R^2^ = 0.488). PE was positively associated with self-reported caring behaviors after adjustment for the demographic and professional covariates (B = 0.52; 95% CI, 0.42–0.63; *p* < 0.001), whereas SE was not independently associated with self-reported caring behaviors (B = 0.01; 95% CI, −0.02 to 0.03; *p* = 0.653). Diploma-qualified nurses reported lower caring behaviors scores than bachelor’s-qualified nurses (B = −0.40; 95% CI, −0.69 to −0.12; *p* = 0.006); the associations with gender and nursing experience were no longer statistically significant. Adding the SE × PE interaction in Model 3 increased the explained variance by a further 1.18 percentage points (ΔR^2^ = 0.0118), but this increment did not reach statistical significance according to the HC3 Wald test (F [1, 193] = 3.43; *p* = 0.066). The interaction estimate was negative but not statistically significant (B = −0.022; 95% CI, −0.046 to 0.001; *p* = 0.066). The final model explained 52.7% of the variance in caring behaviors (adjusted R^2^ = 0.498) and was significant overall (HC3 robust Wald F [12, 193] = 13.66; *p* < 0.001). PE remained positively associated with self-reported caring behaviors (B = 0.49; 95% CI, 0.38–0.60; *p* < 0.001), while diploma qualification remained associated with lower scores relative to a bachelor’s degree (B = −0.34; 95% CI, −0.61 to −0.07; *p* = 0.015) (Table 4).

### 3.3. Nonlinearity and Sensitivity Analyses

**Figure 3:**
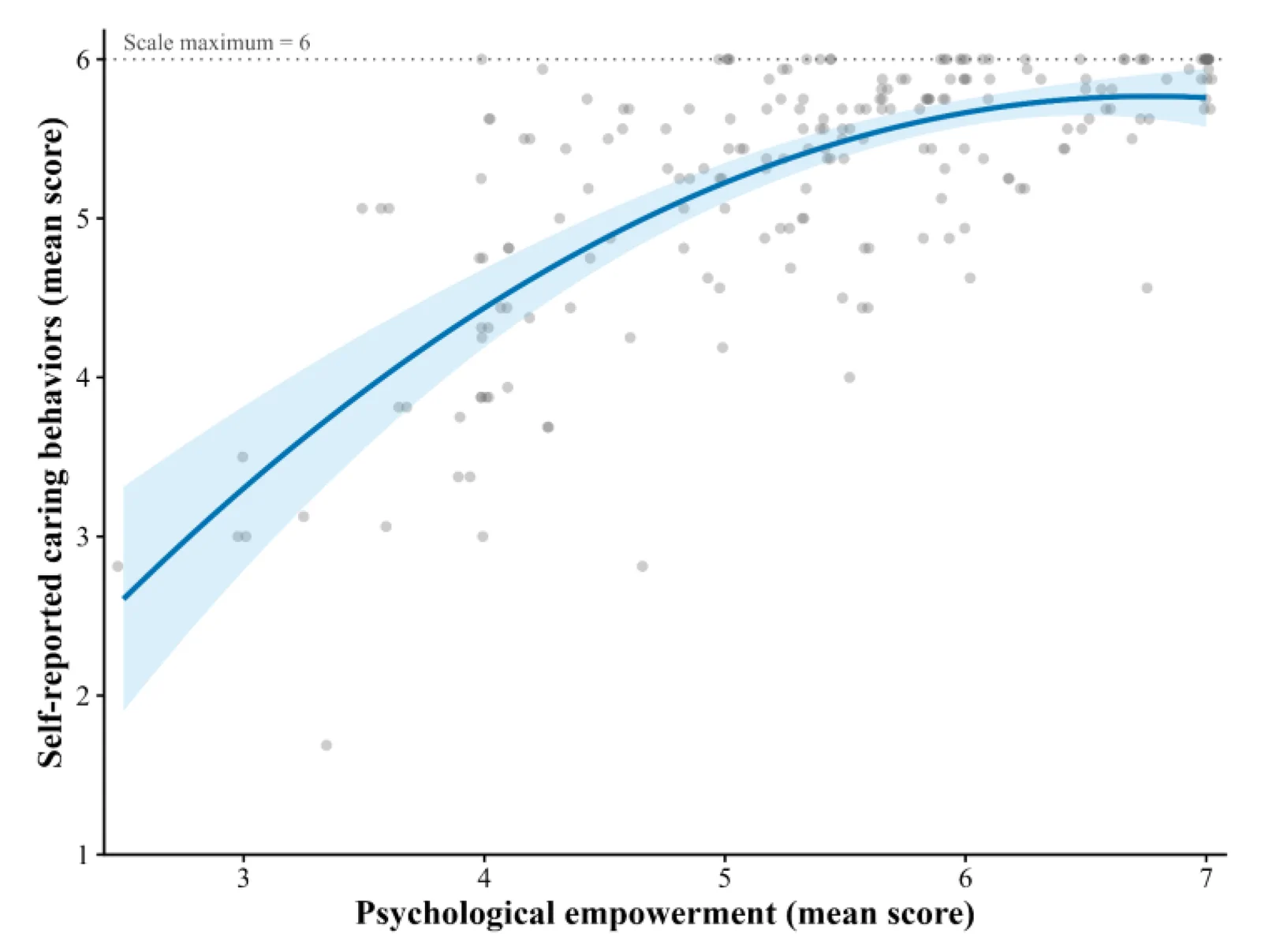
Adjusted nonlinear association between PE and self-reported caring behaviors. Points represent observed scores. The solid line represents average adjusted predictions from the quadratic regression model, and the shaded band represents the pointwise HC3 robust 95% confidence interval. Predictions were averaged across participants’ observed SE values and demographic and professional covariate profiles and were restricted to the observed PE range. The dotted horizontal line indicates the maximum possible caring-behaviors mean score of 6.

In the secondary quadratic model, the squared SE and PE terms were jointly significant (HC3 Wald F [2, 191] = 12.02; *p* < 0.001). The squared PE coefficient was negative (B = −0.17; 95% CI, −0.24 to −0.10; *p* < 0.001), whereas the squared SE coefficient was not statistically significant (*p* = 0.969). The adjusted curve showed a marked increase in self-reported caring behaviors across low and moderate PE levels, followed by a plateau as PE approached the upper end of its observed range (Figure 3). The quadratic model explained 57.1% of the variance in self-reported caring behaviors (adjusted R^2^ = 0.540). After accounting for the quadratic terms, the SE × PE interaction remained non-significant (B = 0.01; 95% CI, −0.02 to 0.04; *p* = 0.674).

The interaction also remained non-significant in the fractional-logit model (*p* = 0.823) and after excluding 11 observations identified using Cook’s distance greater than 4/N (B = −0.02; 95% CI, −0.04 to 0.00; *p* = 0.099; N = 195). Full results from the quadratic, fractional-logit, and influence analyses are provided in Supplementary Table S2.

### 3.4. Exploratory Mediation Analysis

In the exploratory mediation analysis, SE was positively associated with PE (path a: B = 0.13; 95% CI, 0.09–0.16; *p* < 0.001), and PE was positively associated with self-reported caring behaviors after controlling for SE (path b: B = 0.52; 95% CI, 0.42–0.63; *p* < 0.001). The total association between SE and self-reported caring behaviors was statistically significant (path c: B = 0.07; 95% CI, 0.04–0.10; *p* < 0.001), whereas the direct association after accounting for PE was small and not statistically significant (path c′: B = 0.01; 95% CI, −0.02 to 0.03; *p* = 0.653). The estimated indirect effect through PE was 0.066 (bootstrap 95% CI, 0.045–0.091), with the confidence interval excluding zero. The mediator model explained 36.0% of the variance in psychological empowerment, while the outcome models explained 23.3% of the variance before and 51.5% after PE was included (Supplementary Table S4). Because all variables were measured concurrently, this analysis identifies a statistical indirect effect but does not establish temporal ordering or causal mediation.

## 4. Discussion

This study found that structural and psychological empowerment were positively associated with self-reported caring behaviors, with the strongest association observed for PE. These findings suggest that nurses’ perceptions of meaning, competence, self-determination, and impact are associated with how they evaluate their own caring behaviors. This interpretation is supported by Spreitzer’s model, which emphasizes these four dimensions as central to individuals’ experience of empowerment [11]. It can also be understood in relation to Watson’s theory, which emphasizes meaningful and intentional engagement in nurse-patient relationships [1].

SE was positively correlated with PE. This is consistent with previous research [26]. Kanter’s Structural Empowerment Theory and Spreitzer’s PE model can explain this relationship [11]. Kanter emphasizes that access to organizational resources, information, support, and opportunities creates empowering workplace conditions, whereas Spreitzer conceptualizes empowerment as an individual psychological experience reflected in meaning, competence, self-determination, and impact. Therefore, nurses who perceive greater access to empowering workplace structures may also be more likely to experience a strong sense of PE in their professional roles.

Opportunity received the highest SE rating, whereas formal power received the lowest. This pattern is consistent with findings from previous studies among nurses [27,28]. From the perspective of Kanter’s theory, the higher opportunity rating may indicate that nurses perceive greater access to professional growth, learning, and skill development, which are important elements of an empowering work environment. In contrast, the lower rating of formal power may reflect the nature of nursing roles, in which empowerment may be experienced more through opportunities for professional development and clinical practice than through formal organizational authority. Therefore, differences across these dimensions may represent variation in how SE is experienced rather than an overall lack of empowerment.

SE was positively associated with self-reported caring behaviors in the bivariate analysis, but this association was not significant after accounting for PE and demographic and professional covariates. This finding is consistent with previous research [29], where SE was indirectly and positively associated with caring behaviors through compassion fatigue among ICU nurses in China. Although the nature of this association differs from that observed in the present study, these findings suggest that empowering workplace conditions are relevant to nurses’ caring behaviors.

Regarding PE, meaning and competence were rated higher than self-determination and impact. This is consistent with previous studies among nurses in Saudi Arabia [30,31]. From the perspective of Spreitzer’s PE model, the higher ratings of meaning and competence suggest that nurses perceive their work as personally meaningful and feel confident in their ability to perform their roles effectively.

The positive association between PE and self-reported caring behaviors is consistent with previous research [32]. However, this association does not establish whether empowerment perceptions precede caring behaviors or reflect nurses’ evaluations of their caring practice. Reverse directionality is also possible; nurses who evaluate their caring behaviors more favorably may also report greater competence, meaning, and impact. This finding can be interpreted through Spreitzer’s PE model, as nurses who perceive greater meaning and competence in their work, experience greater self-determination, and perceive that their actions have an impact may be more engaged in caring practice.

Caring behaviors clustered toward the upper end of the scale. Although the unadjusted analysis identified a negative interaction between structural and psychological empowerment, the adjusted analysis did not provide sufficient evidence for the hypothesized moderating role of PE. Interaction terms were also nonsignificant across the sensitivity models, while the quadratic analysis identified curvature in the psychological empowerment–caring behaviors association. The adjusted analysis did not support H4, which proposed that PE moderates the relationship between SE and self-reported caring behaviors. Previous research has examined alternative relationships between SE and PE, including indirect pathways through PE [16]. Organizational culture and climate have been linked to empowerment among nurse managers [33], while SE has been associated with job stress and burnout among nurses [34]. An association between nurse-to-patient ratios and employee empowerment has also been reported [35]. These findings suggest that organizational conditions warrant consideration when interpreting empowerment-related associations. Organizational climate, workload, nurse-to-patient ratios, burnout, and leadership quality were not measured in this study and may contribute to residual confounding.

The exploratory mediation analysis identified a positive indirect association between SE and self-reported caring behaviors through PE. SE was positively associated with PE, and PE remained positively associated with self-reported caring behaviors after controlling for SE and the demographic and professional covariates. The direct association between SE and self-reported caring behaviors was small and not statistically significant after accounting for PE. This pattern is compatible with PE as a possible intermediate pathway, although the concurrent measurement of all variables prevents establishing temporal ordering or causal mediation. The exploratory finding does not change the primary moderation result. Therefore, longitudinal studies are needed to examine whether changes in SE precede changes in PE and subsequent self-reported caring behaviors.

The three associations among the three constructs are consistent with previous evidence. This study extends the evidence through adjusted analyses and the identification of nonlinear and exploratory associations in a Saudi hospital sample.

### 4.1. Implications for Nursing

The study findings have implications for nursing and clinical practice. The findings suggest that the work environment and nurses’ psychological experience of empowerment warrant consideration in nursing management research. Access to information, resources, support, and professional development, alongside meaning, competence, self-determination and impact, could be examined as potential targets in future intervention studies. However, the present findings do not establish that increasing SE or PE improves caring behaviors, and the adjusted analysis did not support the hypothesized moderation. Longitudinal or experimental research is needed before recommending these approaches to improve caring practice.

### 4.2. Limitations and Recommendations for Future Study

Several limitations should be considered. The cross-sectional design limits causal inferences regarding the relationships among SE, PE, and self-reported caring behaviors. The exploratory mediation analysis cannot establish temporal ordering or causal mediation. All primary variables were measured through the same self-administered survey at a single time point, creating the possibility of common-method variance and social-desirability bias. Accordingly, the caring-behaviors outcome represents nurses’ self-reported caring behaviors rather than independently observed clinical practice. Scores were also concentrated near the upper scale boundary: the mean was 5.26 on a 1–6 scale, the median was 5.56, and 17.0% of participants had the maximum possible mean score. This possible ceiling effect may have limited discrimination at higher levels and affected the estimated associations. The association between nursing education and self-reported caring behaviors should be interpreted cautiously because only 28 participants held a diploma, multiple covariate comparisons were examined, and residual confounding remains possible. Convenience sampling from a single healthcare setting limits the generalizability of the findings across Saudi Arabia. Nurses with greater professional engagement may have been more willing to participate, introducing possible selection bias. Future studies should use longitudinal designs with repeated measurements and incorporate patient-reported, observer-rated, or other independent measures of caring behaviors.

The pooled medical-unit reference category included medical, psychiatric, obstetric/gynecological, and pediatric services and therefore did not represent a homogeneous clinical environment. Although aggregation avoided unstable estimates from very small unit-specific samples, it may have obscured differences among these specialties and introduced residual within-group heterogeneity. Clinical-unit coefficients should therefore be interpreted as comparisons with the pooled reference category rather than with a single uniform clinical setting.

## 5. Conclusions

In this sample of nurses from a single Saudi hospital, SE and PE were positively correlated with self-reported caring behaviors among nurses. SE was also positively associated with PE. Adjusted and sensitivity analyses provided insufficient evidence for the hypothesized moderating role of PE. The study extends existing correlational evidence by testing this interaction and identifying nonlinear and exploratory indirect associations in secondary analysis. These findings require longitudinal evaluation using independent measures of caring behaviors and do not establish causal relationships or intervention effectiveness.

## Figures and Tables

**Table 1:** Participant demographic and professional characteristics.

Characteristic	Value
**Gender**	
Female	174 (84.5)
Male	32 (15.5)
**Age (years)**	
Mean (SD)	32.43 (9.23)
**Marital status**	
Single	111 (53.9)
Married	76 (36.9)
Divorced	13 (6.3)
Widowed	6 (2.9)
**Highest nursing education**	
Diploma	28 (13.6)
Bachelor’s degree	162 (78.6)
Master’s degree	13 (6.3)
Doctorate	3 (1.5)
**Nursing experience (years)**	
Mean (SD)	8.25 (7.81)
**Clinical unit**	
Medical unit	101 (49.0)
Surgical ward	18 (8.7)
Critical care and emergency	64 (31.1)
Outpatient/clinic	23 (11.2)

N = 206. Categorical values are n (%); percentages use available responses for each variable and may not sum to 100 because of rounding. Continuous values are mean (SD). SD = standard deviation. No missing values were present in the reported variables. Before regression modeling, clinical units were consolidated to avoid unstable estimates from sparsely represented categories. The original distribution was as follows: medical ward (n = 24), surgical ward (n = 18), ICU/critical care (n = 35), emergency (n = 29), psychiatric/mental health (n = 2), obstetrics/gynecology (n = 69), pediatrics (n = 6), and outpatient/clinic (n = 23). The pooled medical-unit category combines medical ward, psychiatric/mental health, obstetrics/gynecology, and pediatrics; the critical care/emergency category combines ICU/critical care and emergency. Surgical ward and outpatient/clinic were retained separately. The pooled medical-unit category was used as the reference group in the regression analyses.

**Table 2:** Descriptive statistics and internal consistency of SE, PE, and self-reported caring behaviors.

Scale/Subscale	No. of Items	N	Possible Range	Observed Range	Mean (SD)	Median [IQR]	Reliability Coefficient (95% CI)
**SE: six-subscale total**	19	206	6–30	7.50–30.00	20.71 (4.24)	20.54 [18.00–23.40]	0.93 (0.91–0.94)
Opportunity	3	206	1–5	1.00–5.00	3.81 (0.92)	4.00 [3.00–4.67]	0.80 (0.73–0.85)
Information	3	206	1–5	1.00–5.00	3.61 (0.90)	3.67 [3.00–4.33]	0.82 (0.76–0.87)
Support	3	206	1–5	1.00–5.00	3.41 (0.93)	3.33 [3.00–4.00]	0.84 (0.79–0.89)
Resources	3	206	1–5	1.00–5.00	3.41 (0.85)	3.33 [3.00–4.00]	0.81 (0.74–0.86)
Formal power	3	206	1–5	1.00–5.00	3.05 (0.98)	3.00 [2.67–3.67]	0.89 (0.84–0.92)
Informal power	4	206	1–5	1.00–5.00	3.41 (0.81)	3.38 [3.00–4.00]	0.77 (0.69–0.84)
**Global empowerment (mean)**	2	206	1–5	1.00–5.00	3.34 (1.03)	3.00 [3.00–4.00]	0.90 (0.84–0.94)
**PE (total)**	12	206	12–84	30.00–84.00	64.61 (12.38)	65.00 [55.25–73.00]	0.93 (0.92–0.95)
**PE (mean)**	12	206	1–7	2.50–7.00	5.38 (1.03)	5.42 [4.60–6.08]	—
Meaning	3	206	1–7	2.00–7.00	5.65 (1.21)	6.00 [4.67–6.67]	0.85 (0.80–0.89)
Competence	3	206	1–7	2.67–7.00	5.65 (1.08)	5.67 [5.00–6.33]	0.84 (0.79–0.88)
Self-determination	3	206	1–7	1.67–7.00	5.18 (1.24)	5.17 [4.08–6.00]	0.82 (0.76–0.87)
Impact	3	206	1–7	1.67–7.00	5.06 (1.17)	5.00 [4.33–6.00]	0.85 (0.79–0.89)
**Self-reported caring behaviors (total)**	16	206	16–96	27.00–96.00	84.24 (12.96)	89.00 [79.00–94.00]	0.95 (0.93–0.96)
**Self-reported caring behaviors (mean)**	16	206	1–6	1.69–6.00	5.26 (0.81)	5.56 [4.94–5.88]	—

**Table 3:** Associations among SE, PE, and CBI.

Association	N	Pearson’s r	95% CI	Raw *p*-Value	Bonferroni *p*-Value
SE–CBI	206	0.38	0.25 to 0.49	<0.001	<0.001
SE–PE	206	0.52	0.41 to 0.61	<0.001	<0.001
PE–CBI	206	0.69	0.61 to 0.76	<0.001	<0.001

SE = structural empowerment; PE = psychological empowerment; CBI = caring behaviors; CI = confidence interval. Pearson correlations used complete pairs (N = 206 for each association). All tests were two-sided. CIs are individual, unadjusted 95% Fisher-z intervals. Bonferroni-adjusted *p*-values account for the three planned comparisons. Spearman sensitivity analyses yielded similar associations: SE–CBI, ρ = 0.40; SE–PE, ρ = 0.56; and PE–CBI, ρ = 0.65 (all Bonferroni-adjusted *p* < 0.001).

**Table 4:** Hierarchical regression models of self-reported caring behaviors and the SE × PE interaction.

	Model 1 Covariates only		Model 2 Covariates + SE and PE		Model 3 Model 2 + SE × PE	
Predictor	B (95% CI)	*p*	B (95% CI)	*p*	B (95% CI)	*p*
***Demographic and professional factors***						
Male vs. female	−0.47 (−0.86 to −0.07)	***0.020***	−0.20 (−0.46 to 0.06)	0.133	−0.20 (−0.44 to 0.05)	0.121
Nursing experience, per year	0.02 (0.01 to 0.04)	***0.007***	0.00 (−0.01 to 0.02)	0.601	0.00 (−0.01 to 0.01)	0.794
Married vs. single	0.00 (−0.25 to 0.25)	0.985	0.00 (−0.18 to 0.19)	0.992	0.02 (−0.16 to 0.20)	0.816
Previously married vs. single	−0.14 (−0.75 to 0.46)	0.642	−0.01 (−0.48 to 0.47)	0.983	−0.02 (−0.48 to 0.45)	0.943
Diploma vs. bachelor’s degree	−0.42 (−0.88 to 0.04)	0.075	−0.40 (−0.69 to −0.12)	***0.006***	−0.34 (−0.61 to −0.07)	***0.015***
Postgraduate vs. bachelor’s degree	−0.16 (−0.66 to 0.35)	0.546	−0.22 (−0.60 to 0.17)	0.269	−0.19 (−0.59 to 0.21)	0.361
Surgical ward vs. medical unit	0.17 (−0.25 to 0.59)	0.415	0.00 (−0.27 to 0.27)	0.973	0.01 (−0.26 to 0.28)	0.937
Critical care/emergency vs. medical unit	−0.01 (−0.27 to 0.25)	0.942	−0.01 (−0.21 to 0.19)	0.940	−0.02 (−0.22 to 0.19)	0.884
Outpatient/clinic vs. medical unit	−0.07 (−0.52 to 0.38)	0.759	−0.09 (−0.37 to 0.19)	0.523	−0.06 (−0.32 to 0.20)	0.643
***Empowerment measures***						
Structural empowerment (centered)	—	—	0.01 (−0.02 to 0.03)	0.653	0.01 (−0.02 to 0.04)	0.370
Psychological empowerment (centered)	—	—	0.52 (0.42 to 0.63)	**<** ***0.001***	0.49 (0.38 to 0.60)	**<** ***0.001***
***Interaction***						
SE × PE	—	—	—	—	−0.022 (−0.046 to 0.001)	0.066
***Model fit***						
Observations, n	206		206		206	
R^2^	0.102		0.515		0.527	
Adjusted R^2^	0.060		0.488		0.498	
Robust Wald F (df1, df2)	1.40 (9, 196)		13.24 (11, 194)		13.66 (12, 193)	
Robust model *p*		0.191		**<** ***0.001***		**<** ***0.001***
Change in R^2^ from preceding model	—		0.4137		0.0118	
Added-block HC3 Wald F (df1, df2)	—		65.40 (2, 194)		3.43 (1, 193)	
Added-block HC3 Wald *p*		—		**<** ***0.001***		0.066

Outcome: self-reported caring behaviors mean score (1–6). Values are OLS unstandardized B (95% CI), with HC3 heteroskedasticity-robust CIs and two-sided *p*-values using residual-df t distributions. Model 1 includes gender, nursing experience, marital status, education, and clinical unit. Model 2 adds SE and PE. Model 3 adds SE × PE. All models use the same complete-case sample; 0 observations were excluded for missing model variables. No influence-based exclusions were applied. SE and PE are centered at 20.712 and 5.384, respectively. In Model 3, the SE and PE coefficients refer to the mean of the other empowerment measure. Intercepts are fitted but omitted from display. Reference categories: female, single, bachelor’s degree, and medical unit. Previously married combines divorced/widowed; postgraduate combines master’s/doctorate. Medical unit combines medical ward, psychiatric/mental health, OB/GYN, and pediatrics; critical care/emergency combines ICU and emergency. Overall model tests are joint HC3 Wald F tests of all non-intercept coefficients. R^2^ and adjusted R^2^ are OLS fit measures. Changes in R^2^ compare each model with the immediately preceding model on identical observations. The added-block HC3 Wald tests jointly assess SE and PE in Model 2 and the SE × PE interaction in Model 3. Bold italic *p*-values indicate *p* < 0.05; no multiplicity adjustment is applied. Interaction estimates use three decimals to retain precision near zero. A dash indicates not applicable. SE, structural empowerment; PE, psychological empowerment; CI, confidence interval.

## Data Availability

The data presented in this study are available on request from the corresponding author. The data are not publicly available due to ethical restrictions.

## Supplementary Materials

The following supporting information can be downloaded at https://www.mdpi.com/article/10.3390/healthcare14213634/s1; Figure S1: Regression diagnostic plots; Table S1: Model fit and formal diagnostics for conventional OLS moderation models; Table S2: Sensitivity analyses of the adjusted moderation model: influence, quadratic curvature, and bounded outcome modeling; Table S3: Univariable associations of demographic and professional characteristics with caring behaviors; Table S4: Exploratory mediation analysis of the association between structural empowerment and caring behaviors through psychological empowerment.

## Abbreviations

The following abbreviations are used in this manuscript:

SE	Structural empowerment
PE	Psychological empowerment
CBI	Caring Behaviors Inventory
CI	Confidence interval
IQR	Interquartile range
SDs	Standard deviations
OLS	Ordinary least-squares
