# Supplementary material for: Self-Reported Caring Behaviors, Structural Empowerment, and Psychological Empowerment Among Nurses: A Cross-Sectional Study
Source: Healthcare (Basel). 2026 Oct 27;14(21):3634. doi: 10.3390/healthcare14213634 (PMC13763345; doi:10.3390/healthcare14213634)
Supplement: Supplementary file 1 [file healthcare-14-03634-s001.zip › healthcare-4599953-supplementary.pdf]

Supplementary materials

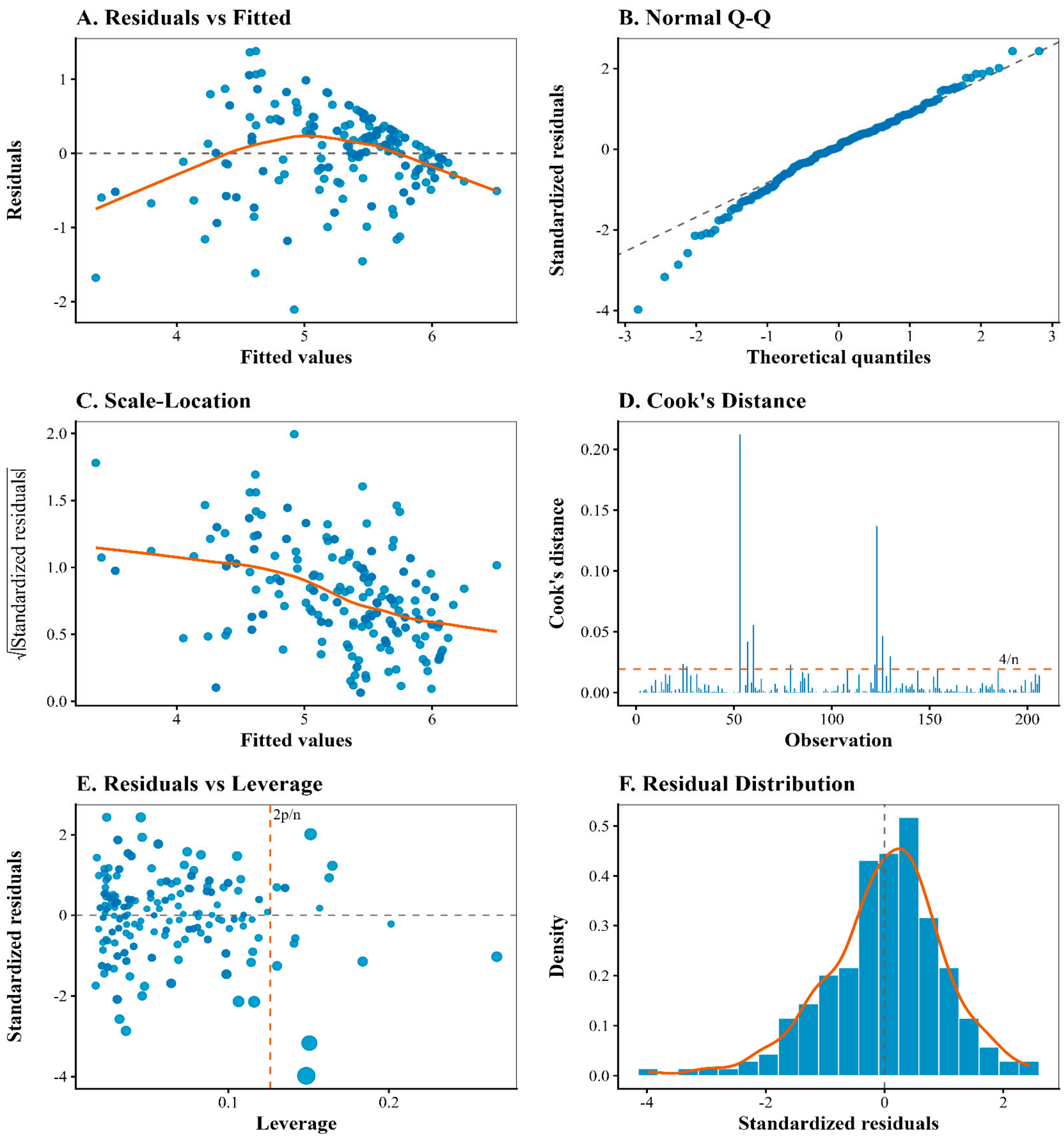

Diagnostics are based on the adjusted moderation model predicting caring behaviors (N = 206). Dashed lines mark zero residuals, the Q-Q reference line, Cook's distance of 4/n, and leverage of 2p/n. p includes the intercept; bubble size reflects Cook's distance. Cutoffs are screening guides; no observations were excluded based on these diagnostics.

Figure S1: Regression diagnostic plots

Table S1
Model fit and formal diagnostics for conventional OLS moderation models.

**Model diagnostics**	**Unadjusted moderation model**	**Adjusted moderation model**
N	206	206
R-squared	0.498	0.527
Adjusted R-squared	0.491	0.498
Overall conventional F-statistic	66.92	17.92
F df1	3	12
F df2	202	193
F p-value	<0.001	<0.001
Studentized Breusch–Pagan	30.92	35.12
BP df	3	12
BP p-value	<0.001	<0.001
Rainbow (functional form)	1.69	1.56
Rainbow df1	103	103
Rainbow df2	99	90
Rainbow p-value	0.005	0.016
Shapiro–Wilk W	0.97	0.98
Shapiro p-value	<0.001	0.005
Anderson–Darling A	1.84	1.08
AD p-value	<0.001	0.008
Maximum-adjusted GVIF	1.23	1.30
Mean-adjusted GVIF	1.17	1.15

Outcome: caring behaviors mean score. Both conventional OLS models include centered structural empowerment (SE), centered psychological empowerment (PE), and SE x PE, on the same complete-case sample. Adjusted covariates: gender, nursing experience, marital status, education, clinical unit. The overall F test tests all non-intercept coefficients jointly, not specifically moderation. No HC3 correction is used in this table. Studentized Breusch–Pagan tests constant error variance using the model regressors. Rainbow uses the central 50% after ordering by fitted values to assess functional form; heteroskedasticity can affect its calibration. Shapiro–Wilk and Anderson–Darling assess residual normality; their residual-based p-values are approximate because fitted residuals are not independent observations. They are complementary diagnostics, not independent evidence. Adjusted GVIF = GVIF^(1/(2 x term df)); one-df terms equal sqrt(VIF). Values summarize all non-intercept model terms, including the interaction; ordinary VIF cutoffs of 5 or 10 do not apply directly to this scale. df = degrees of freedom. Diagnostic p-values are exploratory and unadjusted for multiplicity. Interpret with the diagnostic figure; non-significance does not prove assumptions hold. Conventional F inference may be unreliable with unequal variance; primary regression inference uses HC3.

Table S2
Sensitivity analyses of the adjusted moderation model: influence, quadratic curvature, and bounded outcome modeling.

	**Primary OLS: HC3**		**Fractional logit: HC3**		**Cases excluded OLS: HC3**		**Quadratic OLS: HC3**	
**Predictor / effect / statistic**	**B / effect (95% CI)**	**p**	**B / effect (95% CI)**	**p**	**B / effect (95% CI)**	**p**	**B / effect (95% CI)**	**p**
Intercept	5.40 (5.23, 5.56)	<0.001	2.21 (1.91, 2.52)	<0.001	5.36 (5.20, 5.52)	<0.001	5.53 (5.35, 5.71)	<0.001
Structural empowerment (centered)	0.01 (-0.02, 0.04)	0.370	0.01 (-0.04, 0.06)	0.781	0.02 (-0.01, 0.04)	0.167	0.00 (-0.03, 0.03)	0.832
Psychological empowerment (centered)	0.49 (0.38, 0.60)	<0.001	0.88 (0.69, 1.08)	<0.001	0.45 (0.36, 0.54)	<0.001	0.48 (0.37, 0.59)	<0.001
SE x PE interaction	**-0.02 (-0.05, 0.00)**	**0.066**	**0.00 (-0.03, 0.04)**	**0.823**	**-0.02 (-0.04, 0.00)**	**0.099**	**0.01 (-0.02, 0.04)**	**0.674**
Structural empowerment squared	—		—		—		-0.00 (-0.00, 0.00)	0.969
Psychological empowerment squared	—		—		—		-0.17 (-0.24, -0.10)	<0.001
Male vs female	-0.20 (-0.44, 0.05)	0.121	-0.22 (-0.55, 0.11)	0.187	-0.23 (-0.46, -0.01)	0.043	-0.18 (-0.40, 0.04)	0.112
Nursing experience (per year)	0.00 (-0.01, 0.01)	0.794	0.00 (-0.02, 0.03)	0.959	-0.00 (-0.01, 0.01)	0.784	0.00 (-0.01, 0.01)	0.939
Married vs single	0.02 (-0.16, 0.20)	0.816	0.00 (-0.31, 0.32)	0.981	0.07 (-0.10, 0.24)	0.417	0.00 (-0.17, 0.18)	0.960
Previously married vs single	-0.02 (-0.48, 0.45)	0.943	-0.11 (-0.89, 0.67)	0.787	0.17 (-0.08, 0.41)	0.180	-0.04 (-0.51, 0.43)	0.867
Diploma vs bachelor's degree	-0.34 (-0.61, -0.07)	0.015	-0.49 (-0.90, -0.09)	0.017	-0.31 (-0.52, -0.09)	0.006	-0.30 (-0.56, -0.04)	0.023
Postgraduate vs bachelor's degree	-0.19 (-0.59, 0.21)	0.361	-0.23 (-0.88, 0.41)	0.482	-0.05 (-0.31, 0.21)	0.686	-0.11 (-0.48, 0.26)	0.557
Surgical ward vs medical unit	0.01 (-0.26, 0.28)	0.937	0.09 (-0.39, 0.56)	0.724	0.05 (-0.17, 0.27)	0.663	0.08 (-0.14, 0.31)	0.458
Critical care/emergency vs medical unit	-0.02 (-0.22, 0.19)	0.884	-0.10 (-0.45, 0.26)	0.593	0.05 (-0.13, 0.23)	0.604	-0.05 (-0.24, 0.15)	0.647
Outpatient/clinic vs medical unit	-0.06 (-0.32, 0.20)	0.643	-0.19 (-0.63, 0.24)	0.379	0.08 (-0.12, 0.28)	0.453	-0.11 (-0.36, 0.15)	0.413
N	206		206		195		206	
Influential cases excluded	0		0		11		0	
R-squared (OLS only)	0.527		—		0.535		0.571	
Adjusted R-squared (OLS only)	0.498		—		0.504		0.540	
Interaction change in R-squared	0.0118		—		0.0063		0.0006	
Quadratic terms: joint HC3 Wald F	—		—		—		12.02	<0.001

Values are coefficient/effect (95% CI), with p-values in separate columns. B for the three OLS models is in CBI points; fractional-logit coefficients are on the logit-mean scale and are NOT directly comparable to OLS B. Fractional logit models (CBI-1)/5 using quasi-binomial logit with HC3 covariance and asymptotic normal inference; exact endpoints are retained. OLS inference uses HC3 and residual-df t distributions. All models adjust for gender, nursing experience, marital status, education, and clinical unit. Exclusion removes 11 cases flagged once by Cook's distance > 4/N (0.01942) in the primary model. Quadratic and fractional models retain the full primary sample. Centering is unchanged. OLS R-squared is not supplied for fractional logit; values across different samples are not direct fit comparisons. Each interaction change in R-squared omits only SE x PE on the same respective sample. Joint quadratic Wald test uses F(2,191). Exact maximum CBI score: 35/206 (17.0%). A dash means not applicable. SE = structural empowerment; PE = psychological empowerment.

Table S3
Univariable associations of demographic and professional characteristics with caring behaviors.

Characteristic	N	B	95% CI	p-value	Overall p-value
**Gender**	206				0.003
Female (reference)		Reference			
Male		-0.459	-0.761 to -0.158	0.003	
Age (per year)	206	0.009	-0.003 to 0.021	0.130	0.130
Nursing experience (per year)	206	0.016	0.002 to 0.030	0.026	0.026
**Marital status**	206				0.609
Single (reference)		Reference			
Married		0.120	-0.118 to 0.359	0.321	
Previously married		0.040	-0.358 to 0.437	0.844	
**Nursing education**	206				0.242
Bachelor's degree (reference)		Reference			
Diploma		-0.275	-0.601 to 0.051	0.098	
Postgraduate		-0.107	-0.525 to 0.310	0.613	
**Clinical unit**	206				0.881
Medical unit (reference)		Reference			
Surgical ward		0.068	-0.343 to 0.479	0.745	
Critical care and emergency		-0.032	-0.289 to 0.225	0.807	
Outpatient/clinic		-0.125	-0.496 to 0.246	0.507	

Outcome: caring behaviors mean score. Each predictor was examined in a separate univariable linear regression. B = unstandardized coefficient; CI = confidence interval. Continuous coefficients are per one-year increase; categorical coefficients compare each level with its stated reference. N is the number with available outcome and predictor values. Overall p-values are F-tests of the entire predictor against an intercept-only model on the same observations. Coefficient p-values are two-sided t-tests; CIs use conventional OLS standard errors. Previously married combines divorced/widowed; postgraduate combines master's/doctorate. Medical unit (reference) combines medical ward, psychiatric/mental health, OB/GYN, and pediatrics. Other clinical groups are surgical ward, critical care/emergency, and outpatient/clinic. These analyses are exploratory, with unadjusted p-values; they do not define automatic inclusion or exclusion criteria for the moderation model.

Table S4
Exploratory mediation analysis of the association between structural empowerment and caring behaviors through psychological empowerment.

**Path, effect, or model statistic**	**B or statistic (95% CI)**	**p-value**
Path a: SE to PE	0.13 (0.09 to 0.16)	***<0.001***
Path b: PE to caring behaviors, controlling for SE	0.52 (0.42 to 0.63)	***<0.001***
Total association (c): SE to caring behaviors	0.07 (0.04 to 0.10)	***<0.001***
Direct association (c-prime): SE to caring behaviors, controlling for PE	0.01 (-0.02 to 0.03)	0.653
Indirect association (a x b): SE through PE	0.066 (0.045 to 0.091)	—
Model information		
Observations, n	206	
R-squared: PE mediator model	0.360	
R-squared: total-association outcome model	0.233	
R-squared: direct-association outcome model	0.515	

Outcome: self-reported caring behaviors mean score (1–6). SE = structural empowerment; PE = psychological empowerment; B = unstandardized OLS coefficient; CI = confidence interval. All paths adjust for gender, nursing experience, marital status, nursing education, and clinical unit and use the same complete-case sample. SE and PE were centered at 20.712 and 5.384, respectively. Component-path CIs and two-sided p-values use HC3 heteroskedasticity-robust standard errors and residual-df t distributions. The indirect association is a x b; its 95% percentile bootstrap CI uses 5,000 participant resamples. No p-value is reported for the indirect association; evidence against a null indirect association is assessed by whether its bootstrap CI excludes zero. Previously married combines divorced/widowed; postgraduate combines master's/doctorate. Medical unit combines medical ward, psychiatric/mental health, OB/GYN, and pediatrics; critical care/emergency combines ICU and emergency. Reference categories are female, single, bachelor's degree, and medical unit. Because SE, PE, and caring behaviors were measured concurrently, this exploratory analysis quantifies a cross-sectional indirect association and does not establish temporal or causal mediation.
